# A joint analysis of transcriptomic and metabolomic data uncovers enhanced enzyme-metabolite coupling in breast cancer

**DOI:** 10.1038/srep29662

**Published:** 2016-07-13

**Authors:** Noam Auslander, Keren Yizhak, Adam Weinstock, Anuradha Budhu, Wei Tang, Xin Wei Wang, Stefan Ambs, Eytan Ruppin

**Affiliations:** 1 Center for Bioinformatics and Computational Biology and the Department of Computer Science, University of Maryland, College Park 20742, Maryland, USA; 2 The Blavatnik School of Computer Science, Tel Aviv University, Tel Aviv 69978, Israel; 3 Liver Carcinogenesis Section, Laboratory of Human Carcinogenesis, Center for Cancer Research, National Cancer Institute, Bethesda, Maryland, USA; 4 Molecular Epidemiology Section, Laboratory of Human Carcinogenesis, National Cancer Institute, National Institutes of Health, Bethesda, Maryland, USA; 5 The Sackler School of Medicine, Tel Aviv University, Tel Aviv 69978, Israel

## Abstract

Disrupted regulation of cellular processes is considered one of the hallmarks of cancer. We analyze metabolomic and transcriptomic profiles jointly collected from breast cancer and hepatocellular carcinoma patients to explore the associations between the expression of metabolic enzymes and the levels of the metabolites participating in the reactions they catalyze. Surprisingly, both breast cancer and hepatocellular tumors exhibit an increase in their gene-metabolites associations compared to noncancerous adjacent tissues. Following, we build predictors of metabolite levels from the expression of the enzyme genes catalyzing them. Applying these predictors to a large cohort of breast cancer samples we find that depleted levels of key cancer-related metabolites including glucose, glycine, serine and acetate are significantly associated with improved patient survival. Thus, we show that the levels of a wide range of metabolites in breast cancer can be successfully predicted from the transcriptome, going beyond the limited set of those measured.

The use of metabolomic profiling in cancer provides an additional layer of pathophysiological knowledge beyond genomic data, and is an important tool for the identification of cancer biomarkers both *in vitro* and *in vivo*^1^,^2^, leading to the discovery of key oncometabolites^3^,^4^. While non-targeted metabolomics methods have generated highly important insights^3^, most mechanistic links are still revealed by targeted metabolomics approaches, typically covering less than 200 predefined metabolite^5^.

The systematic investigation and contextualization of metabolomic data can be considerably enhanced by the integration of other data types such as transcriptomics, thus linking known metabolites and genes via their shared metabolic reactions and pathways. Previous studies have integrated these data types in a variety of biological systems including the study of plant nutritional responses^6^, *E.coli* stress response^7^, the identification of new biomarkers in type 2 diabetes^8^ and of biomarkers associated with cancer progression and outcome^9^,^10^,^11^. Several such integrative studies have investigated the metabolic differences between cancer types and subtypes^12^,^13^,^14^,^15^,^16^.

An additional fundamental usage of these high-throughput data has been to study cellular regulation via the identification of reactions and pathways controlled by either *metabolic* or *transcriptional (hierarchical)* regulation, as previously been done in yeast^17^ as well as the characterization of condition dependent regulatory signatures^18^. The flux in a metabolically regulated reaction is mainly a function of its substrates and products levels, while the flux of a transcriptionally regulated reaction is mainly controlled by the expression level of the enzyme catalyzing it. Here we set to study the associations between substrate and product levels and the expression levels of the enzyme encoding their associated reaction. Despite the increased accumulation of metabolomic data, no previous study has systematically integrated large-scale transcriptomic and metabolomic signatures collected from the same tissue samples in cancer to comprehensively study the associations between genes and metabolites on a network-scale level. Thus, we chart these relations with the analysis of matched non-cancerous versus cancer samples via a new machine learning-based pipeline designed to (1) identify reactions manifesting significant enzyme-metabolites associations, and then (2) use this information to predict the actual metabolite levels associated with such reactions from the expression of the genes encoding the enzymes catalyzing them. Such a predictor can go beyond the currently rather limited coverage of measured metabolites and obtain estimations of the levels of additional metabolites whose levels are strongly associated with the enzymes catalyzing the reactions in which they are involved.

## Results

**Figure 1:**
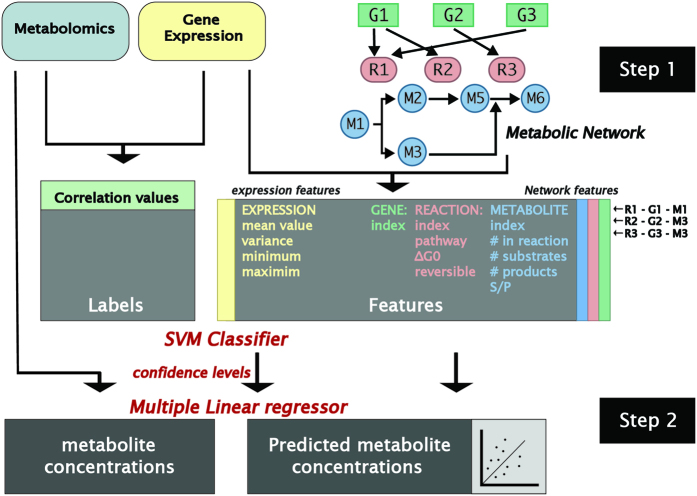
(**A**) The prediction pipeline: Step (1) A classifier predicting RGM triplets that are significantly associated: using Metabolomic and transcriptomic data to identify genes and metabolites that are connected via a metabolic reaction and are significantly associated with each other. This is obtained via building an RGM SVM classifier where each instance represents a unique RGM triplet and whose output is a confidence level signifying whether the gene expression and metabolite levels are significantly positively or negatively associated across all samples (Methods). Step (2) A regressor predicting metabolite levels from gene expression in a sample-specific manner: Confidence levels predicted by the classifier for each RGM triplet in the first step are utilized together with the expression and network features to build a generalized multiple linear regression predictor of metabolite levels from the pertaining enzymes’ gene expression levels (Methods).

We analyzed recently published data of joint transcriptomic and metabolomic measurements across 105 noncancerous and cancerous breast cancer (BC) clinical samples^19^. To systematically study the association between genes and metabolites we utilized the manually curated human metabolic network Recon1, in which genes are mapped to metabolites through their catalyzed metabolic reactions^20^ (Fig. 1A). Out of 162 cytoplasmic metabolites and 1393 genes that could be mapped to the metabolic network, 1107 pairs were found to be connected to each other via a biochemical reaction; that is, the gene’s enzyme product catalyzes a reaction that consumes or produces the metabolite (such gene-metabolites (GM) are termed *connected* herewith). The correlation between the metabolomic and transcriptomic levels of each of these pairs was computed across both non-cancerous and cancer samples, as well as for each of these conditions separately. We find that more than 50% of the gene (enzyme) – metabolite pairs sharing a joint reaction are significantly associated with each other across samples when analyzing the combined non-cancerous and cancer cohorts (FDR-corrected Spearman correlation P-value < 0.05). A smaller number of significant associations is found for each of these two cohorts alone, but while cancer samples show a significantly high number of significant gene-metabolite associations versus random, noncancerous samples do not show this trend (empirical P-values < 0.001 and 0.279 respectively, Table 1, Methods). These results point to a marked increase in the level of enzyme-metabolite associations in cancer versus healthy tissues.

We next aimed to systematically predict enzyme-metabolite associations on a genome-wide level. To this end we developed a two-step pipeline that (1) first performs a binary prediction of which reaction-gene-metabolite associations are statistically significant across the whole human metabolic network. (2) Second, it then utilizes these predicted associations to build a generalized regression predictor of the actual metabolite levels in a given sample from its gene expression data for any reaction in the human metabolic network (Fig. 1).

**Figure 2:**
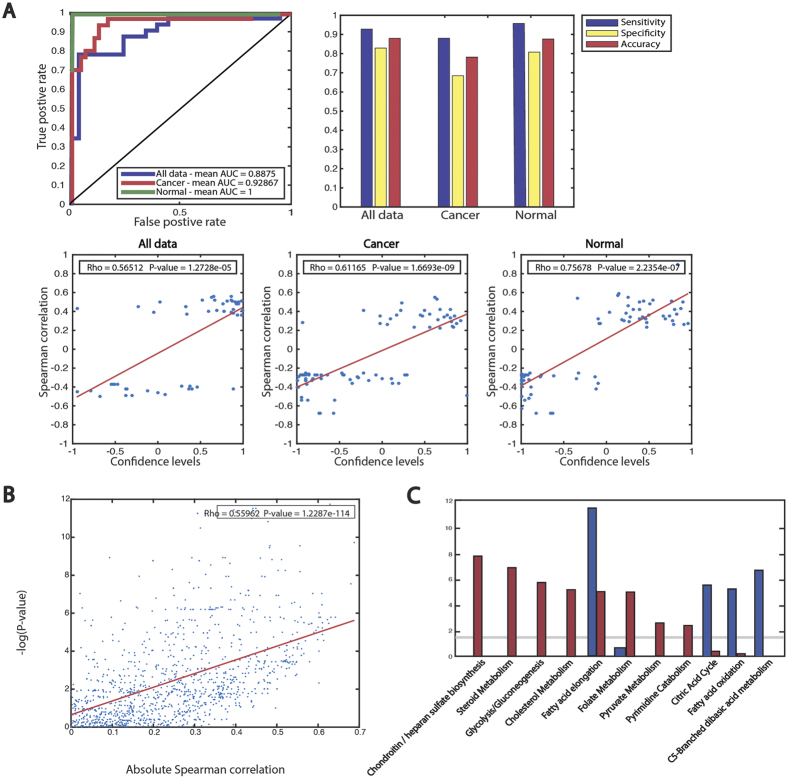
(**A**) Top panels describe the mean AUC of the RGM predictors for all the breast data together and for cancer and noncancerous samples separately. The sensitivity, specificity and accuracy levels of the different classifiers are indicated as well. Bottom panels display the correlation between the confidence levels of the RGM predictions and the gene-metabolite correlations actually measured in the data, for the three cases studied here (see main text). Confidence levels range between −1 and 1 where 1 represents a highly confident positive association and −1 a highly confident negative association. (**B**) Scatter plot representing the association, for all genes, between (1) the absolute Spearman correlation between gene and metabolites associated with it across all samples (x-axis) and (2) the magnitude of the differential expression of that gene between noncancerous and cancer samples (y-axis). (**C**) Pathways that are predicted to be regulated in healthy (red) and cancer (blue) samples. The dashed line represents a hyper-geometric significance threshold of 0.05 (FDR-corrected for multiple hypotheses testing).

For the first, binary classification task, we built a Support Vector Machine (SVM) classifier whose goal is to identify reaction-gene-metabolite (RGM) triplets whose gene and metabolite (connected to the same reaction) exhibit a significant (positive or negative) association. The classifier utilizes gene expression and network features for each RGM triplet while the correlation coefficient between gene and metabolite for each such triplet is used as the classification label. (Fig. 1, Methods). We evaluated the classifier’s performance, termed the *RGM predictor*, via a 5-fold cross validation procedure, using the instances where both the genes’ expression and metabolites levels were measured. The resulting classifier has high prediction accuracy, both when applied to all samples together and when applied to the noncancerous and cancer samples separately (mean AUC = 0.88, 1 and 0.92 respectively, Fig. 2A). Remarkably, a highly significant correlation is obtained between the confidence levels that the SVM classifier assigns to an RGM triplet and the strength of the gene expression-metabolites correlation observed in the measured data (Spearman *ρ* > 0.62, P-value < 1.7e-13 for all three tests described above, Fig. 2A, Supplementary Figure S1).

We applied the RGM classifier described above to predict RGM associations in a genome wide manner and chart a global map of reaction-enzyme-metabolite associations in breast cancer versus healthy samples. We find that the products are more positively correlated with the expression of their associated genes than reaction substrates (one-sided Wilcoxon Rank-sum P-value = 1.19e-69). Similar results are obtained for the breast cancer and healthy cohorts separately (Supplementary Table S1). We next constructed a gene-metabolite bipartite graph, whose nodes are composed of genes and metabolites, and edges connect gene and metabolites sharing joint reactions whose levels where found to be associated in a statistically significant manner (Methods). We find that the cancer bipartite network contains substantially more high degree nodes than the healthy tissue network (Supplementary Figure S2). Remarkably, we find that highly connected genes in the cancer network are significantly associated with three metabolic pathways: Fatty acid activation, Glycolysis/Gluconeogenesis and Extracellular transport, all with low hyper-geometric enrichment P-values, while in the noncancerous network we observe that the Extracellular transport pathway is the only significantly enriched pathway (Supplementary Table S2). The highly connected metabolites, however, are similar between the noncancerous and cancer graphs (hyper-geometric enrichment P-value < 1e-20). The metabolites appearing in the intersection of the two graphs are mainly amino acids (alanine, cysteine, glycine and serine), phosphate and sugars (Supplementary Table S3). We find that the absolute Spearman correlation of gene-metabolite pairs in the data is markedly correlated with the magnitude of the gene’s differential expression across all samples (Spearman *ρ* = 0.55, P-value = 1.22e-114, Fig. 2B), testifying that differentially expressed genes tend to participate in strongly correlated gene-metabolite pairs.

Analyzing the genome-wide predictions of gene-metabolite associations that we obtained, we find a greater amount of significant associations in cancer compared to healthy tissue, as previously observed in the analysis of the raw data. There is only a small overlap between the associations predicted in the healthy and cancer cohorts, providing another indication that cancer metabolism is extensively altered from its healthy state (Supplementary Figure S3), with many enzyme-metabolite interactions being uniquely established in each state. Pathway enrichment analysis summarizing the classifier’s predictions reveals that these associations lie in different pathways in cancer vs healthy samples (FDR-corrected hypergeometric P-value < 0.05, Supplementary Table S4, Fig. 2C). We find that glycolysis is enriched with such associations in cancer while the citric acid cycle is enriched in the latter in healthy samples, in accordance with the central role of glycolysis in cancer and the Warburg effect^21^. Additional pathways that display such increased gene-metabolite coupling in cancer include glycosaminoglycan pathways (including chondroitin and heparan sulfate biosynthesis and degradation), Cholesterol metabolism and folate metabolism, all previously associated with breast cancer^22^,^23^,^24^,^25^.

Next we built a multiple regression predictor of metabolite levels from the gene expression levels of the genes associated with them in the human metabolic network. This second predictor receives the predicted confidence levels in these associations obtained from the previous RGM predictor as inputs (Methods). Applying this regression analysis to all 162 metabolites that were measured across the 105 clinical breast cancer and noncancerous samples reveals an overall moderate but significant correlation between measured and predicted metabolite levels (Spearman correlation *ρ* = 0.33, P-value < 1e-200, for measured metabolites). Overall, 92 of the 162 metabolites (56.8%) show a significant correlation (FDR-corrected Spearman correlation P-value < 0.05) between measured and predicted values across all 105 samples. 77 of 105 samples (73.3%) show a significant correlation between measured and predicted metabolite levels across all 162 measured metabolites (FDR-corrected Spearman correlation P-value < 0.05, Supplementary Figure S4 and Supplementary Tables S5 and S6). For each metabolite, the regression performance (for predicting its level across samples) significantly correlates with its strongest RGM-predicted gene-metabolite association (Spearman correlation *ρ* = 0.71, P-value < 2.6e-25), demonstrating that, as expected, the levels of metabolites that are strongly associated with their enzyme’s expression can be better predicted. Highly predictable metabolites include key amino acids that have been implicated in cancer such as glycine, serine and threonine as well as key glycolytic metabolites (D-glucose-6-phosphate, D-fructose-6-phosphate). Applying the regression to the noncancerous and breast cancer samples separately, a similar overall prediction performance is observed (Spearman correlation *ρ* = 0.3219, *ρ* = 0.3653 for noncancerous and cancer, respectively; P-value < 7.6626e-162 for both cases).

**Figure 3:**
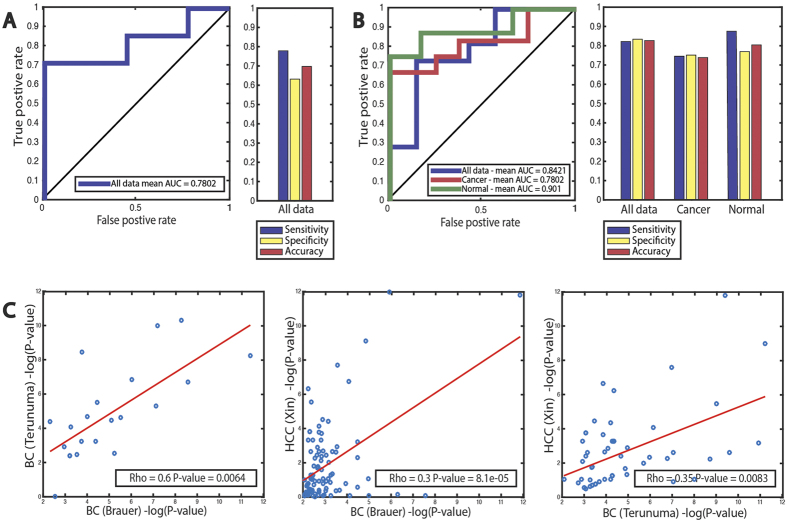
(**A**) The mean AUC of the RGM predictors for the Brauer BC dataset. The sensitivity, specificity and accuracy levels of the different classifiers are indicated as well. (**B**) The mean AUC of the RGM HCC predictors for all the data together and for cancer and healthy cohorts separately, and the sensitivity, specificity and accuracy levels. (**C**) A scatter plot describing the correlation between pathway enrichment p-values among the three datasets, when all significantly enriched pathways in the two datasets were considered (hyper-geometric p-value < 0.05). The left most panel displays the latter for the two BC datasets, and the middle and right most panels compare the HCC values to each of the BC datasets.

To explore whether the enzyme-metabolite associations observed above can be reproduced in an independent breast cancer dataset we repeated the same two steps’ analysis using the data of Brauer *et al*.^26^. This cohort is much smaller in comparison to the previous dataset, having joint transcriptomic and metabolomic measurements across 28 breast cancer samples. Aligning these data with the metabolic network we were able to map 172 cytoplasmic metabolites to 1066 genes and 842 connected gene-metabolite pairs. We then applied the two steps pipeline on this data: (1) we built the SVM RGM classifier to predict RGM associations, resulting in a moderate but significant accuracy (mean AUC = 0.73, Fig. 3B). (2) We built a multiple linear regressor to predict metabolite levels from the gene expression measurements of the genes associated with them. Here we find again a fairly strong correlation between measured and predicted metabolite levels (Spearman correlation *ρ* = 0.54, P-value < 1e-320), and, as before, we observe that the regressor performance significantly correlates with each metabolite’s strongest RGM-predicted gene-metabolite association (Spearman correlation *ρ* = 0.8, P-value < 19 e-20). Examining the associations found at the metabolic pathways level, reassuringly we find that the pathway enrichment P-values assigned to each pathway in this dataset are significantly correlated with the P-values assigned in the larger, previous dataset analyzed above (Spearman *ρ* = 0.6, P-value = 0.0064, Fig. 3C, (Methods, Supplementary Table S4)).

We then sought to explore whether the enhanced enzyme-metabolite coupling found in breast cancer may extend to another cancer type. To this end, we applied the same prediction pipeline and analyses to data from hepatocellular carcinoma (HCC) patients^13^ (such datasets are yet hard to come by). The HCC data is again smaller in its extent compared to the Terunuma data, comprising joint transcriptomic and metabolomic measurements across 27 noncancerous and 29 hepatocellular carcinoma samples. Aligning these data with the metabolic network we were able to map 153 cytoplasmic metabolites, 1219 genes and 1400 connected gene-metabolite pairs. Notably, the HCC data exhibits much fewer significant GM associations −less than 10% of the connected GM pairs are significantly associated (FDR-corrected Spearman P-value < 0.05). Still, analyzing GM associations in the raw data reveals the same trend as that observed in BC data (Supplementary Figure S5), of a marked increase in these associations in cancer. We then built an HCC RGM classifier and evaluated its performance using 5-fold cross validation. It shows high prediction accuracy, both when applied to all samples together and when applied to the healthy and cancer samples separately (mean AUC = 0.84, 0.78 and 0.90 respectively, Fig. 3A). As in the breast cancer case, the confidence level assigned to an RGM triplet significantly correlates with the magnitude of the triplet’s correlation (Spearman correlation *ρ* = 0.26, 0.29, 0.35 and P-value = 0.002, 0.005, 0.0001 for all samples, noncancerous and cancer respectively). We generated a HCC multiple linear regression predictor of metabolite levels from the gene expression levels and applying it to all 153 metabolites across 56 HCC samples. This regressor obtains a significant but rather small overall correlation between measured and predicted metabolite levels (Spearman correlation *ρ* = 0.165, P-value < 2.4e-24, for the measured metabolites). Yet, these findings are quite remarkable given that less than 10% of the reactions show FDR-corrected RGM significant correlations in the original, measured data.

We performed a pathway-level enrichment analysis for the HCC dataset as described before for the BC dataset (Supplementary Table S2). The correlation of the HCC pathway enrichment p-values and the p-values of each of the two breast cancer datasets is lower compared to the correlation between the two BC datasets (Fig. 3C and Supplementary Figure S6) as expected, but still notable. These findings suggest that some increased gene-metabolite couplings are cancer type specific but others may be more generic to cancer. Indeed, we find that fatty acid oxidation is highly coupled in both cancer types for all three datasets, in accordance with recent findings testifying to its role in cancer proliferation^27^,^28^, and that the Glycolysis/Gluconeogenesis pathway displays increased metabolic coupling; the latter that has recently been associated with down-regulation of the p53 tumor suppressor^29^.

**Figure 4:**
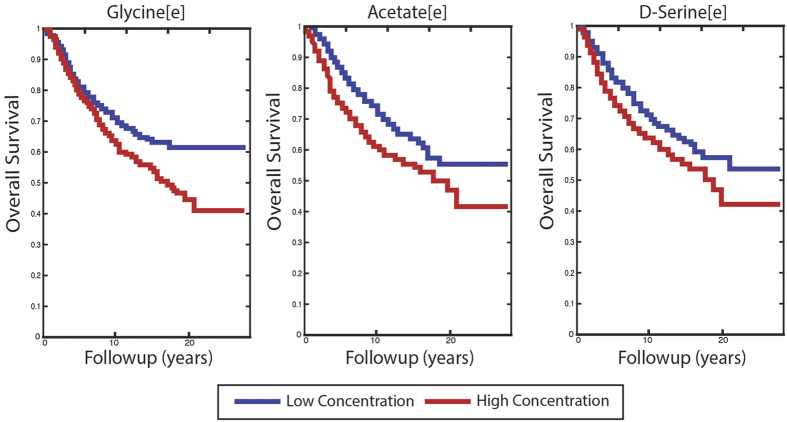
Kaplan-Meier survival plots for extracellular levels of glycine, acetate and serine. The associated FDR-corrected log-rank P-values are 0.002, 5.18e-6 and 8e-4, respectively.

Finally, we applied our metabolite prediction pipeline to another independent large cohort of gene expression data from BC tumors to predict genome-wide metabolite levels in every sample and associate them with the given patients’ survival data^30^. After obtaining patient specific predicted metabolite levels from corresponding gene expression levels (Methods) we examined the association of each metabolite with patients’ survival data in the cohort studied via a Kaplan-Meier survival analysis (Methods). Overall, we find that the predicted levels of 531 metabolites have a significant association with patients’ survival time (FDR-corrected log-rank P-value < 0.05, Supplementary Table S7). Focusing on extracellular metabolites as potential biomarkers, we find that low levels of glycine, serine and acetate are associated with improved survival, in accordance with previous findings regarding these metabolites^31^,^32^,^33^ (Fig. 4, Table 2). Comparing metabolite-based survival results to the survival inferred from the expression of their corresponding genes, we find 168 metabolites whose predicted levels display stronger associations with survival than the associations found for the expression of the genes producing or consuming them (Supplementary Table S9). Finally, we examined which metabolites can significantly differentiate between ER+ and ER− samples. The two leading metabolites are Carbonic acid and CO_2_ (FDR-corrected two-sided Wilcoxon P-value < 1e-150). The levels of these metabolites were not measured in the Terunuma *et al*. BC dataset, but they were predicted to be significantly different between ER+ and ER− samples in this dataset as well (FDR-corrected Wilcoxon rank-sun p-value < 0.05 for both). Interestingly, it was shown that Carbonic anhydrase (CA), which converts carbon dioxide to carbonic acid to regulate cellular pH, is up-regulated in hypoxia in cancer cells^34^,^35^ and is a significant prognostic marker in invasive breast carcinoma^36^.

## Discussion

Many cellular processes are widely considered to be dysregulated in cancer, including metabolism^37^,^38^. The disrupted regulatory processes have many manifestations, such as altered signaling and increased heterogeneity in transcription^39^,^40^,^41^,^42^. Jointly analyzing transcriptomic and metabolomic data revealed increased enzyme-metabolite coupling in both BC and HCC. Although it may appear at first that dysregulation and increased gene-metabolite associations in cancer are conflicting, our findings may actually suggest that they are complementary, occurring at different levels of cellular processing. At the level of transcription, tumor cells exhibit an altered regulatory program dictated by changes in signaling and transcription levels. These changes are followed tightly with corresponding alterations at the metabolite levels in key cancer-related metabolic pathways. Remarkably, examining both BC and HCC cancer types, we find that increased RGM associated genes also show an increased variance in their expression relative to other genes (One-sided Wilcoxon P-value < 4.17e-4 and P-value < 1.18e-7 in BC and HCC respectively, Supplementary information). This finding supports the notion that the heterogeneity in transcription and increased metabolic coupling observed here may be complementary. We hypothesize that increased metabolic coupling in tumors may make them more adaptable to an ever changing harsh environment; the cells’ survival may depend on a quick response at the metabolite level when in metabolically distressed cancer cells. Further, we find that these increased gene-metabolite couplings are not occurring randomly across the metabolic network but are localized to specific pathways, in a pattern that is quite consistent across two different BC datasets.

Taken together our study provides a comprehensive analysis of metabolomic and transcriptomic associations in breast cancer, and highlights metabolic enzyme-metabolite interactions and pathways that are regulated in cancer. We introduce here a method for predicting metabolite levels based on transcriptomics and network properties. As expected, its accuracy increases for metabolites that are strongly associated with their enzyme’s expression. While the association between metabolites and transcription levels is complex due to additional factors in play such as post-transcriptional modifications and protein expression, we still find that many genes and metabolites are directly associated via their relations in the metabolic network. Given that targeted metabolomics is still limited, mostly covering less than 200 metabolite^5^, our prediction pipeline offers news ways for deciphering the role of different metabolites in cancer progression, and for identifying biomarkers for early detection and prognosis. As new and more comprehensive datasets are generated in the future, the prediction pipeline presented here can be further refined to generate more accurate predictions. This in turn may provide additional mechanistic insights to metabolite-enzyme associations in different cancer types and their potential clinical significance.

## Methods

### Studying gene and metabolite associations

All pairwise Spearman correlations coefficients between measured genes (n = 20202) and metabolites (n = 536) across samples were computed. Next, 162 cytoplasmic metabolites and 1393 genes were uniquely mapped to the metabolic network^20^. Metabolites were mapped first based on HMDB and then by KEGG identifiers. Genes were mapped based on their Entrez identifier. The metabolic network was then utilized to identify genes and metabolites that are associated with each other via a biochemical reaction (gene-metabolites (GM) are *associated* if the gene’s enzyme product catalyzes a reaction that consumes or produces the metabolite. To study the extent to which genes and metabolites are associated with each other in a given dataset, we calculated strength of association between connected GM pairs (compared to non-connected pairs) – Given a dataset with C connected GM pairs, a permutation test was performed as follows: gene labels were permuted and C random GM pairs are selected. The number of significant associations (at a significance level of α = 0.05) was counted across the randomized pairs. This procedure was repeated 1000 times and the empiric P-value was computed as 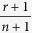 where *r* is the number of times a random permutation achieved a greater number of significant associations than the real data and *n* = 1000.

The tests described were applied to both the BC and HCC datasets, and performed separately for the noncancerous samples, cancer samples and all samples (in each case the correlations were computed across a subset of the samples in the dataset).

In the pathway analysis and survival analysis we extended the set of RGM triplets by adding mapped metabolites from all other cellular compartments, as defined by the human model (n = 434).

### Support Vector Machine (SVM) classification – the RGM predictor

We applied an SVM classifier with a linear kernel and the 14 following features:

Reaction features include:

(1) reaction index in the metabolic network.

(2) an integer value associated with a unique metabolic pathway.

(3) predicted Δ*G*_0_ of each reaction^43^

(4) a binary integer indicating whether the reaction is reversible.

Gene features include:

(5) The gene index in the metabolic network.

(6) The mean value of the gene’s level in the transcriptomic data.

(7) The variance of the gene’s level in the transcriptomic data.

(8),(9) The minimum and maximum values of the transcript level in the transcriptomic data, respectivly.

Metabolite features include:

(10) The metabolite index in the metabolic network.

(11) The total number of metabolites participating in the reaction.

(12) The total number of substrates participating in the reaction.

(13) The total number of products participating in the reaction.

(14) A binary integer indicating whether the metabolite is a substrate or a product.

For the labels we used the significant negative or positive correlations between genes and measured metabolites. Triplets for which both gene and metabolite levels are measured are assigned with the corresponding Spearman correlation and its associated P-value (we here considered each measured metabolite as a cytoplasmic one). RGM triplets with a significant P-value (FDR corrected, α = 0.05) are labeled as positively/negatively associated according to the sign of the correlation coefficient and used as training data. The correlations are computed across the noncancerous and cancer datasets together and apart. Per dataset, the SVM classifier is trained on an equal number of positively and negatively associated RGM triplets. Following training the classifier assigns a confidence level to each RGM triplet (recall that for ~90% of triplets metabolite levels were not measured) in the range [−1, +1], confidence levels close to +1 signifying positively associated triplets and −1 signifying negatively associated triplets. RGM triplets assigned with low confidence values (close to zero) are considered to be non-interactional regulated. Cross-validation was performed by setting aside one fifth of the positively/negatively associated triplets in the training set. The classifier was trained on the remaining four fifths and confidence levels were predicted for the triplets set aside. The classifier’s accuracy was measured by comparing the predicted labels against the known labels.

### The gene-metabolite interactions graph

We represent each significantly correlated gene and metabolite (FDR corrected Spearman correlation, *α* = 0.05) with an edge connecting them. The resulting graphs for noncancerous and cancer samples separately are bipartite graphs in which the degree of each metabolite node is the number of genes that are highly correlated with it and the degree of each gene node is the number of metabolites that are highly correlated with it in either noncancerous or cancer samples. In graphs presented in Figure S2 we show highly connected metabolites with degree *d* > 4.

### Multiple Regression analysis predicting metabolite levels from transcriptomics and network features

A multiple regression analysis is applied between metabolite levels (162 metabolites across 105 samples in the Terunuma *et al*. BC dataset, 172 metabolites across 28 samples for the Brauer *et al*. BC dataset and 153 metabolites across 56 samples in the HCC dataset), and transcriptomic and network features. The features used in this analysis are as follows: for each metabolite *m* we selected two pairs of genes and reactions *GR*_+_(*m*), *GR*_−_(*m*) which are predicted as most positively and negatively correlated with this metabolite respectively, when correlation is defined by the confidence levels of the classifier machine built in the previous step. For each metabolite we identified the 2 RGMs that are predicted as most positively and negatively correlated with it, and used RGM features utilized for their SVM classifier, resulting in 28 features per metabolite as inputs to the multiple linear regressor predictor. Metabolite concentrations are log-normalized and standardized using z-scores. We only reported cases where the predicted confidence level is above 0.5 but the results are robust to different thresholds.

### Pathway Analysis

Two types of pathway analysis were performed – based on the SVM classifier’s predictions and based on GM associations in the raw data. The two types of analysis differed significantly since the classifier’s predictions provided full network coverage at RGM level, whereas the GM associations in the data provided very partial coverage (less than 10% of the GM associations in the metabolic network were measured). When analyzing the classifier’s predictions we first determined which reactions were predicted to be associated with regulated enzyme-metabolite interactions in noncancerous or in cancer. Per reaction, we examined the set of confidence levels assigned to RGM triplets pertaining to that reaction (a reaction typically participates in 6 RGM triplets). A reaction is considered regulated in a condition (cancer/noncancerous) if any of its RGM triplets was assigned with significant confidence level (with an FDR corrected significance level of α = 0.05) for a specific condition. We then performed a hypergeometric enrichment analysis to find pathways enriched with reactions that are regulated in the two conditions (at an FDR corrected significance level of α = 0.05).

When analyzing GM associations in the raw data we lacked sufficient coverage in order to determine which biochemical reactions were interactional regulated. Instead, we examined the set of GM pairs pertaining to each pathway. Spearman correlation P-values denoting GM association strength were computed separately across the noncancerous and cancer conditions. A Wilcoxon rank-sum test (at an FDR corrected significance level of α = 0.05) was then used determine which pathways exhibited a significant shift in interaction regulation by comparing the set of GM association P-values observed in the noncancerous condition against those observed in the cancer condition. Only pathways with 10 or more measured GM pairs were considered for this analysis, in order to insure the stability of the results. We excluded the smaller BC dataset from this analysis since it has only cancer samples.

### Evaluating metabolites association with patients survival

For each sample, we predicted the metabolite levels based on the features from the two RGM triplets predicted as most positively and negatively correlated with it using the classifier generated for the Terunuma *et al*. BC dataset. We then separate the predicted metabolite levels to ‘low’ and ‘high’ by their median level, and calculated the resulting Kaplan-Meier survival log-rank p-value of tumor samples displaying low vs high levels of a given metabolite.

## Additional Information

**How to cite this article**: Auslander, N. *et al*. A joint analysis of transcriptomic and metabolomic data uncovers enhanced enzyme-metabolite coupling in breast cancer. *Sci. Rep.* **6**, 29662; doi: 10.1038/srep29662 (2016).

## Supplementary Material

Supplementary Information

Supplementary Dataset

## Figures and Tables

**Table 1:** A summary of the levels of associations exhibited between connected gene-metabolite pairs in BC and HCC, compared between the noncancerous and cancer conditions.

Test	Statistic used	Breast Cancer		Hepatocellular Carcinoma	
		Noncancerous	Cancer	Noncancerous	Cancer
Enrichment of highly expressed genes with significant GM associations	Hypergeometric P-value	0.998	1.14e-7	0.915	1.6e-7
Strength of association between connected GM pairs (compared to non-connected pairs)	Empiric P-value	0.279	0.001	0.983	0.002

In both datasets the cancer condition exhibits significantly stronger associations than the noncancerous condition.

**Table 2:** The top-ranked metabolites whose extracellular levels is predicted to be negatively associated with patient’ survival.

Metabolite name	Log-rank p-value	Wilcoxon P-value	Fold-change (cancer versus noncancerous)
L-Cysteine	5.11E-05	1.49E-09	4.7
L-Alanine	5.68E-05	3.79E-09	2.24
D-Serine	0.000801	2.64E-10	3.19
Adenosine	0.0025	3.20E-10	5.29
Glycine	0.0027	5.75E-12	3.24
Uracil	0.0031	3.69E-13	7.52
L-Tryptophan	0.0036	3.71E-09	2.15
L-Tyrosine	0.0036	2.01E-06	1.82
L-Proline	0.0041	6.54E-13	3.92
L-Phenylalanine	0.0059	7.33E-07	1.96
Guanosine	0.0096	1.03E-11	2.38
L-Threonine	0.013	1.59E-08	1.97
Inosine	0.015	5.21E-06	1.76
L-Methionine	0.015	2.02E-07	1.97

These metabolites also show a significant difference in their levels in non-cancerous versus cancer samples. The Table indicates the log-rank P-value of the survival analysis, the Wilcoxon P-value of the differential analysis and the fold change of median cancer versus noncancerous metabolite levels (for the full Table see Supplementary Table S8).
