# Supplementary material for: A joint analysis of transcriptomic and metabolomic data uncovers enhanced enzyme-metabolite coupling in breast cancer
Source: Sci Rep. 2016 Jul 13;6:29662. doi: 10.1038/srep29662 (PMC4942812; doi:10.1038/srep29662)
Supplement: Supplementary Information [file srep29662-s1.pdf]

A joint analysis of transcriptomic and metabolomic data uncovers enhanced regulation of enzyme-metabolite interactions in breast cancer

Noam Auslander[3,*,#], Keren Yizhak[1,*,#], Adam Weinstock[1,*], Anuradha Budhu[4], Wei Tang[5], Xin Wei Wang[4], Stefan Ambs[5], Eytan Ruppin[1,2,3,#]

[1] The Blavatnik School of Computer Science, Tel Aviv University, Tel Aviv 69978, Israel
[2] The Sackler School of Medicine, Tel Aviv University, Tel Aviv 69978, Israel
[3] Center for Bioinformatics and Computational Biology and the Department of Computer Science, University of Maryland, College Park 20742, Maryland
[4] Liver Carcinogenesis Section, Laboratory of Human Carcinogenesis, Center for Cancer Research, National Cancer Institute, Bethesda, Maryland
[5] Molecular Epidemiology Section, Laboratory of Human Carcinogenesis, National Cancer Institute, National Institutes of Health, Bethesda, MD 20892;
[*] Equal contribution
[#] Corresponding authors: noamaus@gmail.com; kerenyiz@post.tau.ac.il; eyruppin@gmail.com

Supplementary Figure S1:

Scatter plot of absolute predicted confidence levels achieved by the SVM predictor and the measured gene-metabolite Spearman correlation according to the normal and cancer datasets, together and alone.

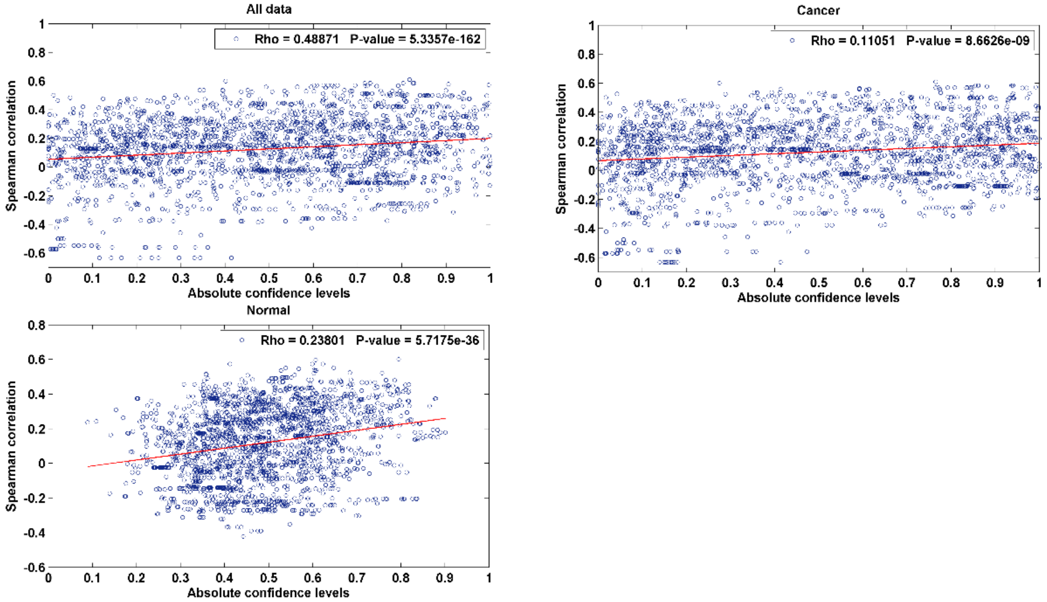

Supplementary Figure S2:

The gene-metabolite interactions graph for normal **(A)** and cancer **(B)** samples. Red nodes represent metabolites, blue nodes represent genes and each edge indicates a significant correlation between gene-metabolite pair.

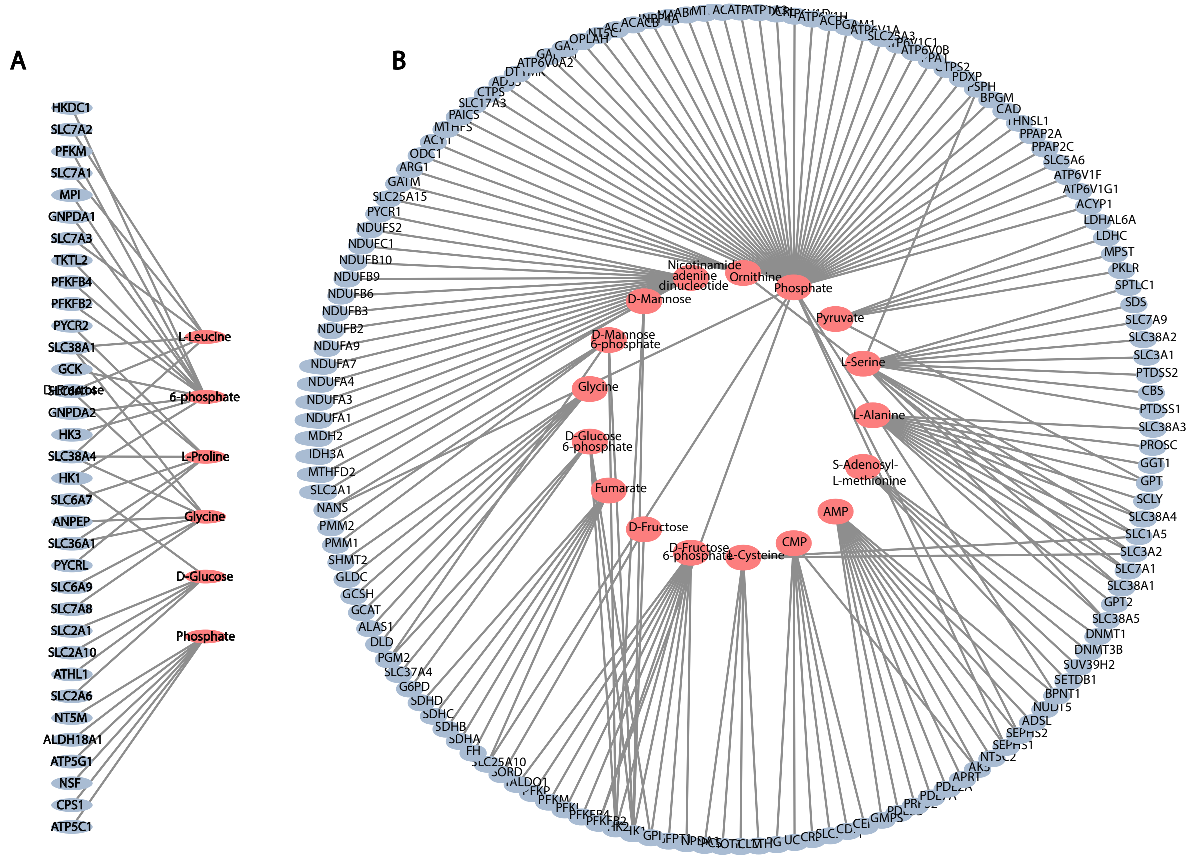

Supplementary Figure S3:

Venn diagram indicating the number of gene-metabolite pairs that are significantly associated with each other in normal and breast cancer samples according to the metabolomics and transcriptomics data.

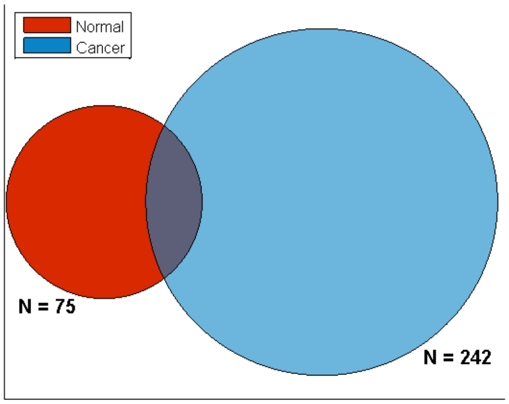

Supplementary Figure S4:

Bar plot of Spearman correlations obtained between measured and predicted metabolite levels for each measured and predicted metabolite across the samples (left) and for each sample across different metabolites (right). The dashed line represents the FDR-corrected significance threshold.

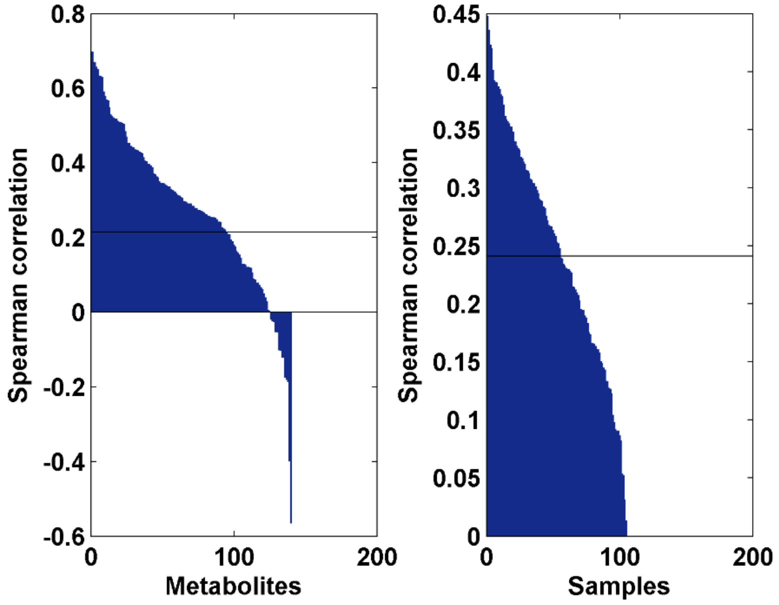

Supplementary Figure S5:

Distribution of gene-metabolite associations in lowly/highly expressed genes across normal samples (blue/red) and cancer samples (green/pink) in BC and HCC measurements. -log(P-value) quantifying the significance of the gene-metabolite pairwise Spearman correlations are shown. The yellow horizontal line denotes a significance level of 0.05. For each of the four conditions the distribution's 90th percentile is represented by a colored horizontal bar and the number of significant gene-metabolite associations is shown in brackets.

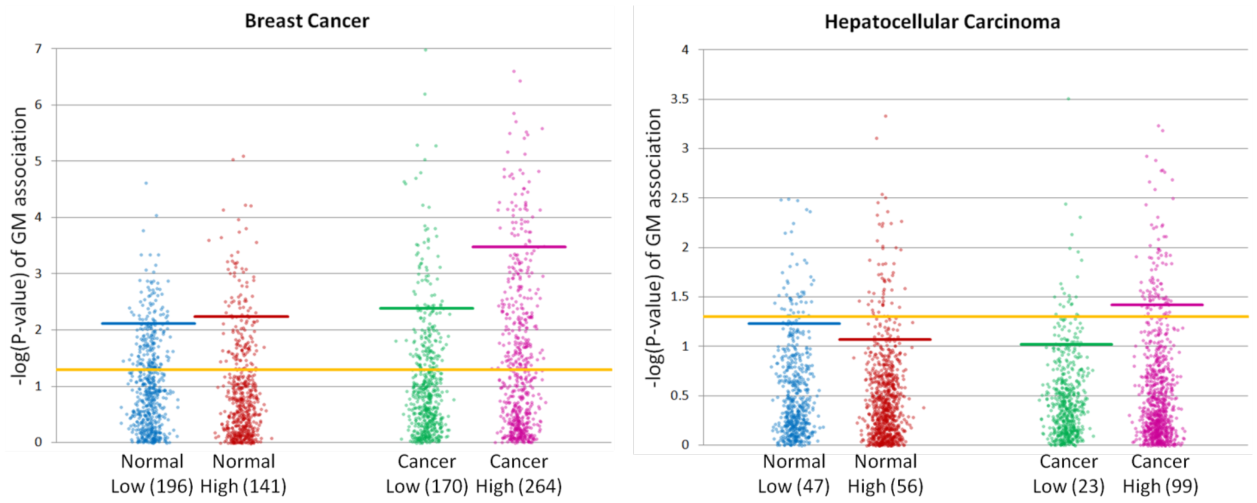

Supplementary Figure S6:

Venn diagram indicating the number of regulated pathways in each of the datasets. Indicated pathways are in the intersection of all three datasets.

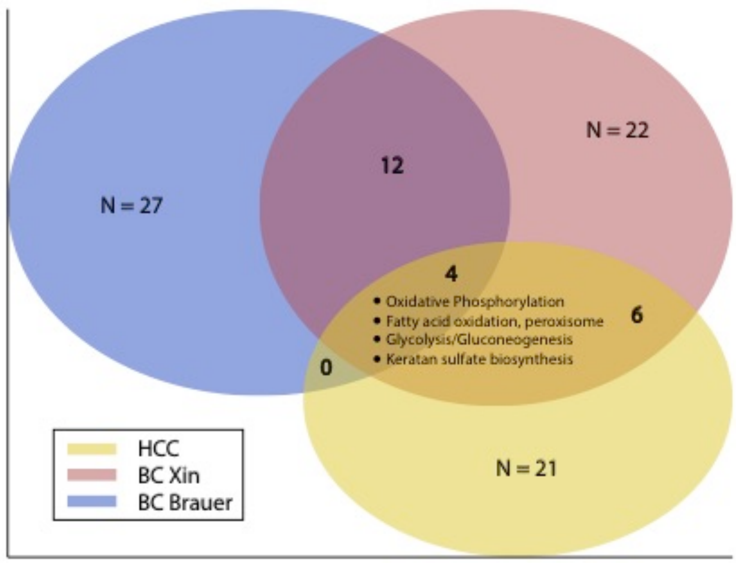

Supplementary Table S1:

One-sided Wilcoxon P-value indicating that products are more positively correlated with their associated genes than with their associated substrates. The results are presented based on both the data and the classifier results when applied to normal and cancer samples separately.

	**Data**	**Classifier**
**Cancer**	0.06	4.9e-131

**Normal**	1e-3	9.9e-15

Supplementary Table S2:

The pathways enrichment for the cancer and normal bipartite network for highly connected metabolites. The p-values indicated are for hyper-geometric enrichment test.

**Cancer**		**Normal**	
**Pathway**	**P-value**	**Pathway**	**P-value**
Transport, Extracellular	1.95E-07	Transport, Extracellular	1.85E-11
Glycolysis/Gluconeogenesis	1.04E-07		
Fatty acid activation	1.65E-05		

Mapping HCC data onto the metabolic network

The HCC data comprises joint transcriptomic and metabolomic measurements across 27 normal and 29 cancerous hepatocellular carcinoma samples. Values were measured for 11228 genes and 469 metabolites. 1219 genes and 168 cytoplasmic metabolites could be mapped onto the metabolic network, yet only 153 metabolites participated in at least one gene-metabolite pair. In total 1400 connected gene-metabolite pairs were found (where both gene and metabolite were measured).
